# Holmium-166 Radioembolization: Current Status and Future Prospective

**DOI:** 10.1007/s00270-022-03187-y

**Published:** 2022-06-21

**Authors:** Martina Stella, Arthur J. A. T. Braat, Rob van Rooij, Hugo W. A. M. de Jong, Marnix G. E. H. Lam

**Affiliations:** Department of Radiology and Nuclear Medicine, UMC Utrecht, Heidelberglaan 100, 3584CX Utrecht, The Netherlands

**Keywords:** Holmium-166, Radioembolization, Dosimetry, SPECT/CT, MRI

## Abstract

Since its first suggestion as possible option for liver radioembolization treatment, the therapeutic isotope holmium-166 (^166^Ho) caught the experts’ attention due to its imaging possibilities. Being not only a beta, but also a gamma emitter and a lanthanide, ^166^Ho can be imaged using single-photon emission computed tomography and magnetic resonance imaging, respectively. Another advantage of ^166^Ho is the possibility to perform the scout and treatment procedure with the same particle. This prospect paves the way to an individualized treatment procedure, gaining more control over dosimetry-based patient selection and treatment planning. In this review, an overview on ^166^Ho liver radioembolization will be presented. The current clinical workflow, together with the most relevant clinical findings and the future prospective will be provided.

## Introduction

Radioembolization, also known as selective internal radiation therapy, is a minimally invasive procedure that combines low-volume embolization and radiation to treat liver cancer. This procedure relies on the principle that hepatic tumors are mainly supplied by hepatic arteries [1]. Thus, radioactive microspheres will predominantly lodge in and around tumorous tissue, sparing healthy liver tissue.

The possibilities to use holmium-166 (^166^Ho) as a potential isotope for the internal radiation therapy of hepatic tumors was first proposed in 1991 by Mumper et al. [2]. Shortly after, Turner et al. [3] investigated single-photon emission computed tomography (SPECT) dosimetry in pigs, while in 2001 Nijsen et al. [4] performed liver tumor targeting in rats by selective delivery of ^166^Ho microspheres. Following these promising results in animals’ studies, in 2010 Smits et al. [5] designed the first phase I human trial to evaluate the safety and toxicity profile of ^166^Ho radioembolization. Since the first publication on ^166^Ho liver radioembolization, there was a growing interest in this treatment possibility, especially in the last years, as suggested by the increasing publications on this topic.

## Holmium-166 Isotope

^166^Ho microspheres were developed at the Department of Radiology and Nuclear Medicine of the University Medical Center Utrecht and were granted a patent in 2007. In 2015, ^166^Ho microspheres received CE mark under the commercial name of QuiremSpheres™ (Quirem BV, Deventer, the Netherlands). A test dose of 250 MBq ^166^Ho microspheres, identical to the ones used for the treatment, received CE mark in 2018 as QuiremScout™ (Quirem BV, Deventer, the Netherlands). A scout dose of ^166^Ho microspheres can be used to safely evaluate the distribution of intra-arterially injected microspheres prior to treatment and eventually adjust it. In terms of clinical application in liver tumors, ^166^Ho microspheres are an alternative to the existing yttrium-90 (^90^Y) devices (either resin or glass).

## Physical and Chemical Properties

^166^Ho microspheres are made of poly-l-lactic acid (PLLA), containing the isotope ^166^Ho. First in the microspheres production process, holmium-165 (^165^Ho) is embedded within the matrix structure of PLLA. Then, a part of ^165^Ho is activated to ^166^Ho by neutron activation in a nuclear reactor. ^166^Ho microspheres characteristics are summarized in Table 1.

**Table 1:** ^166^Ho microspheres characteristics

*T*½	26.8 hours	Microspheres density	1.4 g/cm^3^
*E*_βmax_	1.85 MeV (48.8%)	Relative embolic effect	Medium
*E*_γ_	81 keV (6.6%)	Number of particles	30 million^a^
Range of β particles in tissue (mean and max)	2.5–9 mm	Specific activity (Bq/microsphere)	200–400
Microsphere material	Poly-l-lactic acid	Scout dose	^166^Ho microspheres
Diameter (mean and range)	30 µm, 15–60 µm	Imaging modality	SPECT/MRI

^a^Based on a treatment activity of 10 GBq

## Imaging Possibilities

The radioactive isotope ^166^Ho is a high-energy beta-emitting isotope for therapeutic use, but it also emits primary gamma photons that can be used for SPECT. Furthermore, being a lanthanide, it can be imaged by magnetic resonance imaging (MRI) thanks to its paramagnetic properties, enabling the visualization of its distribution in the liver and quantification of the absorbed tumor dose [6, 7].

### Single-Photon Emission Compute Tomography

Upon decay, the isotope ^166^Ho emits several gamma photons, most of which are 81 keV (abundance 6.6%), 1379 keV (0.9%) or 1581 keV (0.2%). Because ^166^Ho decays to the stable isotope erbium-166 (^166^Er) with a half-life of 26.8 h, there is a time constraint on the imaging procedure; it should be performed within 6 days after administration. On the other hand, immediately after administration, there is an abundance of gamma photons that significantly increases detector dead time (time duration during which the gamma camera is unable to detect a new scintillation after a previous event). In particular, using an acquisition and reconstruction protocol commonly applied in clinical practice, a 20% count loss due to dead time was observed around 0.7 GBq injected activity [8]. Thus, dependent on the amount of administered activity, it is advised to perform post-treatment ^166^Ho SPECT/CT between 2 and 5 days after treatment, aiming at an activity < 0.7 GBq at the time of imaging. To properly image ^166^Ho using SPECT in clinical practice, acquisition parameters are suggested in Table 2.

**Table 2:** Acquisition parameters SPECT

^166^Ho photopeak	Window width	Collimator	Scatter correction	Time per projection
81 keV	15%	MELP	DEW	15 s

*MELP* medium energy low penetration, *DEW* dual energy window

### Magnetic Resonance Imaging

MRI is independent of administered activity; however, because of artifacts, it is limited to tissue without air and metal. MRI has a higher resolution than SPECT/CT.

The presence of Ho (either ^166^Ho or ^165^Ho) accelerates the decay of the ${T}_{2}$ vector of tissue. A linear relationship exists between ${T}_{2}^{*}$ times and Ho concentration [9]. This relationship, called relaxivity (${R}_{2}^{*}$), depends on the strength of the main magnetic field of the scanner and the Ho content. For a best estimate of ${R}_{2}^{*}$ (hence of the local concentration of Ho), a dedicated fitting incorporating the estimated initial amplitude of the free induction decay curve (S_0_-fitting) instead of conventional multigradient echo fitting is suggested [10]. In a retrospective analysis including 14 patients [6], a good correlation was found between the whole liver mean absorbed radiation dose as assessed by MRI and SPECT (correlation coefficient = 0.93; *P* < 0.001), with MRI recovering 89 ± 19% of the delivered activity. A downside of paramagnetic effects of Ho is that susceptibility artifacts obscure gadolinium enhancement on follow-up MRIs, which makes it harder to assess whether treated tumors (or parts) are still enhancing and thus viable. Additionally, the increase in microsphere size, weight percentage or activated fraction of Ho microspheres and field strength can influence sensitivity and detectability of MRI. The impact of these factors on ^166^Ho-MRI imaging was investigated in a dedicated work [11].

## Clinical Workflow

**Fig. 1:**
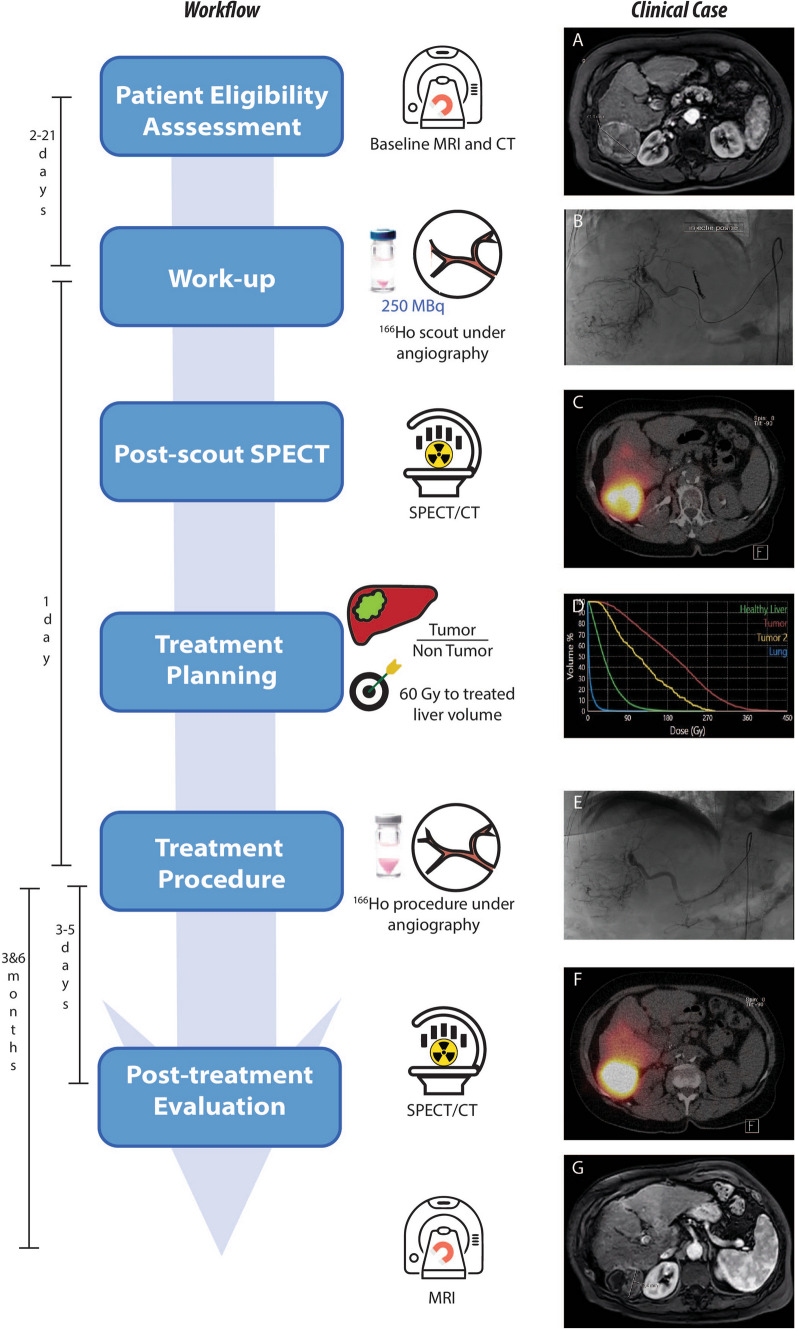
On the left, the steps of the clinical workflow for ^166^Ho liver radioembolization are depicted. On the right, images referring to an exemplary clinical case are reported. A 73 years old female patient diagnosed with hepatocellular carcinoma was referred for ^166^Ho radioembolization. Among others, she presented a lesion in segment 6 with a maximum diameter of 71 mm, as it is possible to see from the baseline MRI reported in panel **A**. During the workup angiography (panel **B**), coil embolization of the segment 4 artery was performed to obtain intrahepatic redistribution. Consequently, activity initially planned for segment 4 was added to the activity injected in the right hepatic artery, for a total of 122 MBq. In the SPECT/CT acquired after the scout procedure and displayed in panel **C**, it is possible to see a clear ^166^Ho uptake in the segment 6, where the tumor lesion was located. No extrahepatic deposition was reported, confirming a successful scout procedure. After having planned the treatment aiming at 60 Gy average absorbed dose to the whole liver (panel **D**), 4116 ^166^Ho MBq was injected into the right hepatic artery (panel **E**). 3 days after the treatment, a SPECT/CT was acquired to visually confirm the good targeting of tumor in segment 6 (panel **F**). Post-treatment dosimetry revealed a good targeting of the tumor, which received a mean dose of 137 Gy, and a safe uptake by the healthy liver, which had a mean absorbed dose of 36 Gy. The MRI acquired 3 months after the treatment (panel **G**) showed a decrease in lesion size of segment 6 from 71 to 42 mm and complete disappearance of contrast enhancement (complete response according to mRECIST)

The clinical workflow for ^166^Ho radioembolization follows similar steps as for radioembolization with other devices. These are summarized in Fig. 1, together with an exemplary clinical case.

### Patient Eligibility Assessment

Indications and contraindications for radioembolization with ^166^Ho-microspheres are the same as for radioembolization with ^90^Y-microspheres [12] and are summarized in Table 3.

**Table 3:** Recommendations and contraindications for radioembolization

Indications	Contraindications
1. Unresectable primary or metastatic hepatic disease with liver-only or liver dominant tumor burden 2. Life expectancy > 3 months 3. An eastern cooperative oncology group (ECOG) status ≤ 2 4. In case of (suspected) cirrhosis; Child–Pugh score ≤ B7 5. Preoperative radioembolization for: (a) Downstaging (b) Bridge to transplant (c) Hypertrophy induction	1. Pretreatment scan demonstrating (a) The potential of > 30 Gy radiation exposure to the lung^a^ (b) Flow to the gastrointestinal tract that cannot be corrected by catheter techniques 2. Limited hepatic reserve (a) Irreversibly elevated bilirubin levels (> 2.0 mg/dl) (b) Reduced albumin (< 3 g/dl) 3. Prior external beam radiation therapy involving the liver in the treatment field of view. Systemic radionuclide treatments are allowed (e.g., ^177^Lu-dotatate) 4. Severe contrast allergy, not manageable or responsive to prophylaxis

^a^Based on manufacturer’s instruction for use

### Workup

During a preparatory angiography, the hepatic vasculature is mapped and injection positions are determined. The use of cone-beam CT is of great additional value in this process and helps to determine if there is extrahepatic contrast enhancement. A small batch of ^166^Ho microspheres with limited activity (200–250 MBq; 60 mg; approximately 3 million ^166^Ho microspheres) can be used as a scout dose. The safety of ^166^Ho scout dose in a clinical setting was demonstrated by Braat et al. [13] in a retrospective study including 82 patients. They did not report any relevant clinical toxicity nor adverse events related to an extrahepatic deposition, which occurred in six patients, after a median follow-up of 4 months.

## Comparison Between ^99m^Tc-MAA and ^166^Ho Scout

Traditionally, radioembolization with ^90^Y requires the use of technetium-99m macroaggregated albumin (^99m^Tc-MAA) as surrogate compound to perform the radioembolization scout procedure. However, ^99m^Tc-MAA differs from the particle used for treatment (either ^90^Y or ^166^Ho microspheres) with respect to shape, size, density and number of injected particles, resulting in a different biodistribution. ^166^Ho radioembolization offers the possibility to use ^166^Ho microspheres for both scout and treatment procedure, reducing the variables among these and theoretically reducing the discrepancy between the planning and the procedure. ^166^Ho scout was shown to have a superior predictive value for intrahepatic distribution in comparison with the commonly used ^99m^Tc-MAA [14]. From the analysis of 71 lesions that received two separate scout dose procedures (^99m^Tc-MAA and ^166^Ho scout), the qualitative analysis showed that ^166^Ho scout was superior to ^99m^Tc-MAA with a median score of 4 versus 2.5 (*P* < 0.001). The quantitative analysis showed significantly narrower 95%-limits of agreement for ^166^Ho scout in comparison with ^99m^Tc-MAA when evaluating lesion absorbed dose (− 90.3 and 105.3 Gy vs. − 164.1 and 197.0 Gy, respectively).

### Post-Scout SPECT

The amount of activity injected during the workup procedure is enough for accurate SPECT/CT quantification, but limited enough not to cause tissue damage in case of shunting to the gastrointestinal organs or the lungs [13]. SPECT images are assessed for the presence of extrahepatic depositions in gastrointestinal organs and for lung shunting, and allow for a re-evaluation of the injection positions. Because the same particles are used, lung shunting can be estimated more accurately, as it was demonstrated by Elschot et al. [15] in 14 patients. Using post-treatment ^166^Ho microspheres SPECT/CT imaging as a reference, pretreatment diagnostic ^166^Ho microspheres SPECT/CT images were significantly better predictors of the actual lung absorbed doses (reference: median 0.02 Gy, range 0.0–0.7 Gy vs. median 0.02 Gy, range 0.0–0.4 Gy). Doses estimated based on ^166^Ho microspheres planar scintigraphy (median 10.4 Gy, range 4.0–17.3 Gy), ^99m^Tc-MAA SPECT/CT imaging (median 2.5 Gy, range 1.2–12.3 Gy) and ^99m^Tc-MAA planar scintigraphy (median 5.5 Gy, range 2.3–18.2 Gy) all overestimated the actual lung absorbed doses.

### Treatment Planning

The anticipated absorbed dose distribution imaged can be assessed using ^166^Ho scout, and treatment planning can be adapted based on this distribution. The current activity calculation for ^166^Ho microspheres is based on a method comparable to the Medical Internal Radiation Dose (MIRD) method. The absorbed dose in Gy delivered by 1 GBq in 1 kg tissue is 15.87 Gy for ^166^Ho, under the assumption of homogenous distribution in the target volume and absorption of all energy within that volume. The formula for the prescribed activity is based on a 60 Gy average absorbed dose to the whole liver:

$${\text{Prescribed}}\,{\text{activity}}\,\left[ {{\text{MBq}}} \right] = {\text{Liver}}\,{\text{weight}}\,\left[ {{\text{kg}}} \right] \times 3781\,\left[ {{\text{MBq/kg}}} \right]$$

According to current instructions for use [16], the average absorbed dose to the perfused volume may exceed 60 Gy (allowing for personalized dosimetry), as long as the average absorbed to the whole liver does not exceed 60 Gy.

### Treatment Procedure

After a successful scout procedure, patients undergo treatment with the administration of the treatment dose in a subsequent treatment procedure. Same-day treatment with ^166^Ho radioembolization is feasible, as proved by van Roekel et al. [17] in 105 patients with a median total procedure time of 6 h and 39 min*.* On the upside this limits complete treatment to one day, on the downside a same-day approach limits possibilities of personalized treatment based on ^166^Ho scout distribution since activity needs to be ordered ahead of time.

### Post-treatment Evaluation

To assess the outcome of the radioembolization procedure, either a SPECT/CT or MRI can be performed. It allows for the quantification of the dose in the compartments of interest, i.e., tumor and heathy liver, and the evaluation of the dose–response effect. For colorectal cancer patients with inoperable, chemorefractory hepatic metastases, a dose–response threshold was found to be 90 Gy, with sensitivity of 100% and specificity of 38% [18]. Dose–response threshold values for patients with hepatocellular carcinoma and patients with liver metastases of neuroendocrine origin are currently under investigation through the analysis of Hepar Primary and Hepar PLuS data, respectively.

A dedicated software package (Q-suite™, Quirem BV, Deventer, The Netherlands) can be used for treatment planning and dose reconstruction for treatment evaluation.

**Fig. 2:**
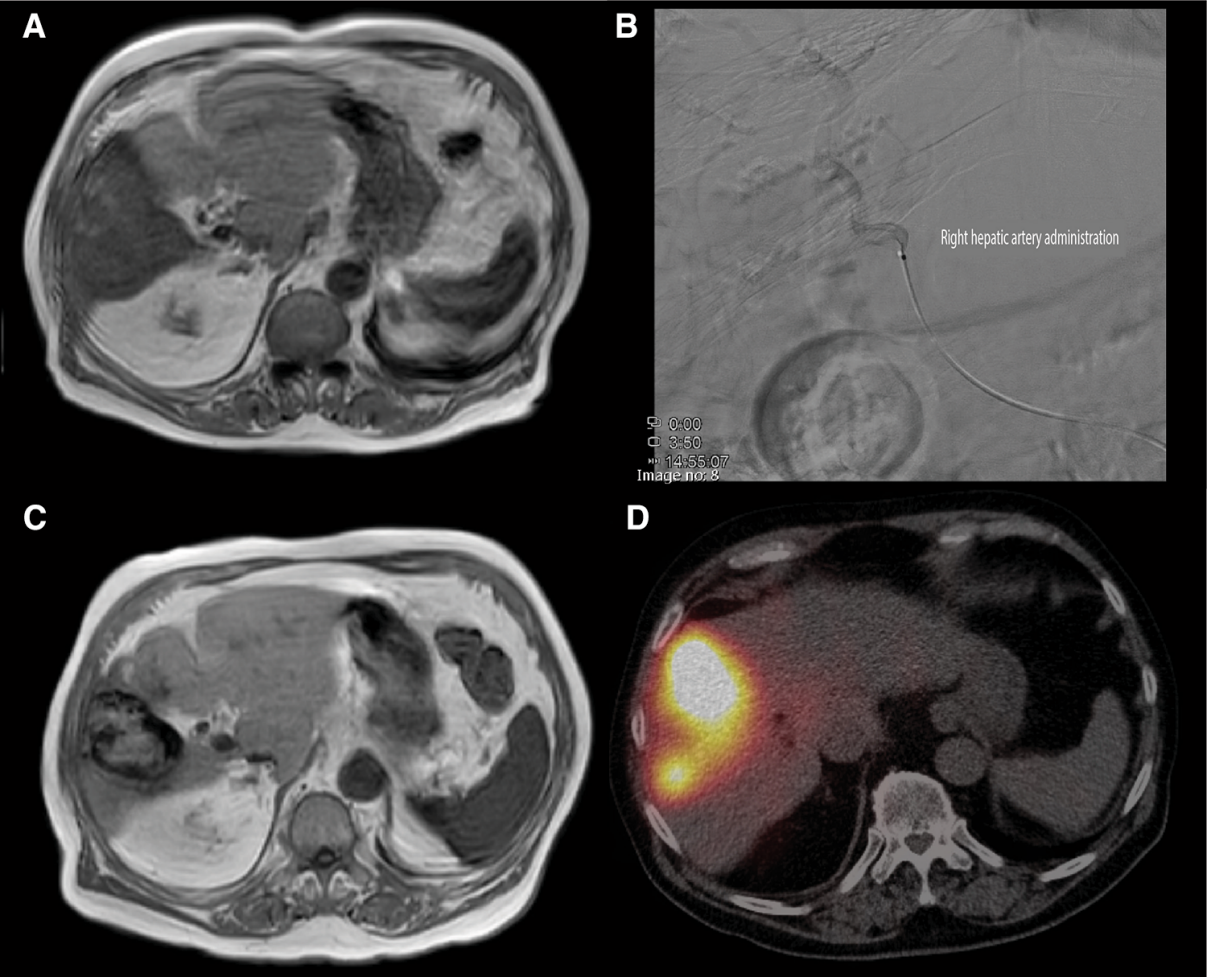
85 year old male diagnosed with hepatocellular carcinoma (HCC). At presentation, contrast enhanced T_1_ MRI (**A**), a solitary hypervascular lesion in segment 5, 6 and 8 with a maximum diameter of 8.1 cm was seen. At tumor board, the patient was considered for first-line SIRT. The ^166^Ho scout procedure consisted of a single injection of 233 MBq of ^166^Ho microspheres in the right hepatic artery (**B**) and subsequent SPECT/CT imaging showed no lung shunt, no extrahepatic deposition of activity elsewhere and visually good tumor targeting. The patient proceeded with ^166^Ho treatment in the afternoon (on the same day), in which 4.3 GBq of ^166^Ho microspheres were administered in the right hepatic artery (**B**). 3 months after treatment, follow-up contrast enhanced T_1_ MRI (**C**), showed a good response reducing its size from 8.1 cm to 5.8 cm and complete response according to mRECIST. Post-treatment SPECT/CT (**D**) 3 days after treatment confirmed the planned high accumulation of particles in the lesion, without extrahepatic deposition of activity (and no lung shunt). At this moment, more than 3 years after treatment, the patient has no signs of recurrent disease on imaging

**Fig. 3:**
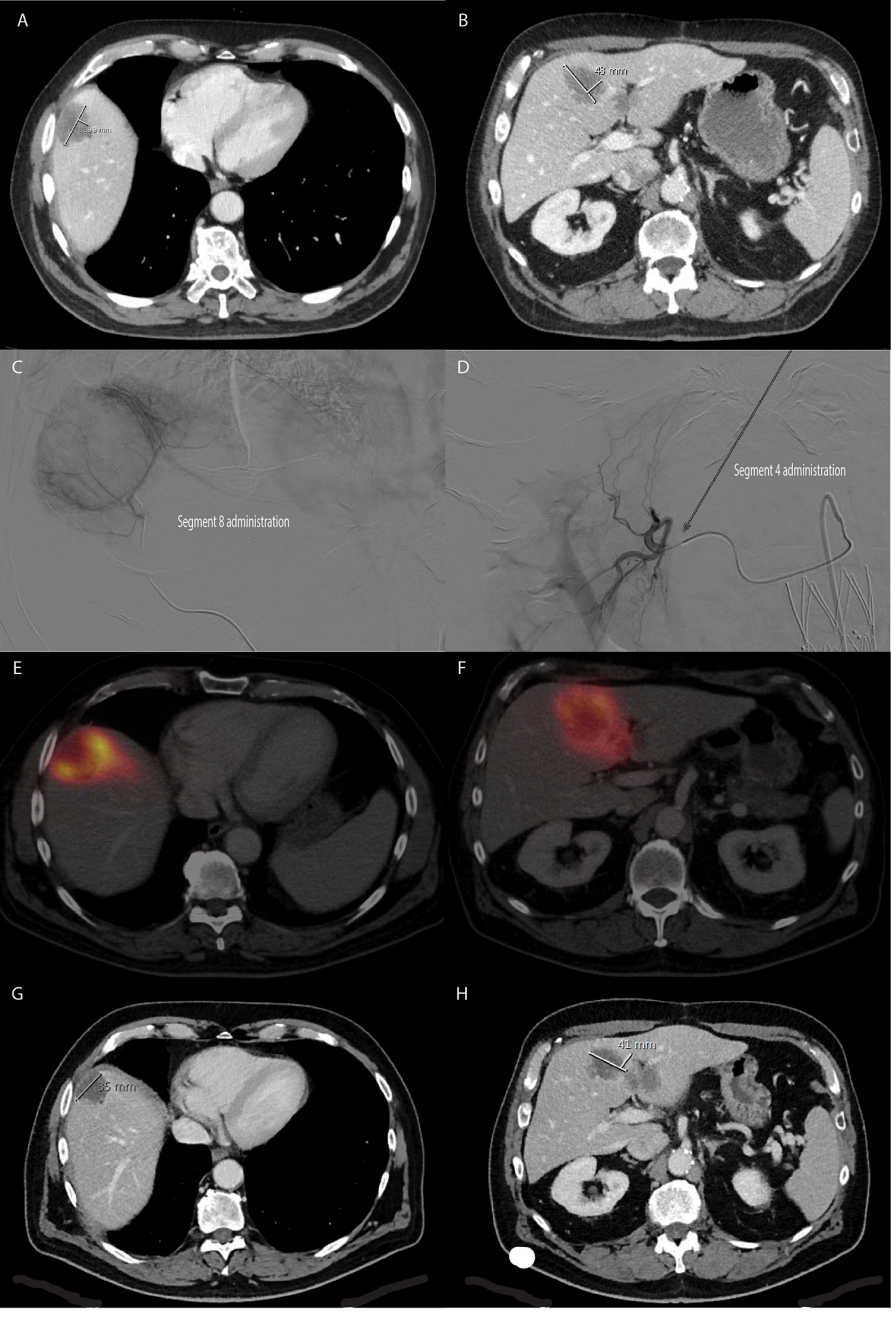
64 year old male diagnosed with intrahepatic metastatic cholangiocarcinoma (ICC), with distinct lesions in segment 8, 4 and a minor lesion on the edge of segment 3/4B (**A** and **B**). At tumor board, he was considered to be eligible for radioembolization treatment with ^166^Ho microspheres, which he received after an uneventful ^166^Ho scout procedure. On the day of treatment a superselective injection of 1.6 GBq (radiation segmentectomy) in segment 8 (**C**) and segmental injection of 0.8 GBq in segment 4 (**D**) was executed. Post-treatment SPECT/CT showed a good accumulation of particles around the tumor in segment 8 (**E**) and segment 4 (**F**). Contrast enhanced CT (**G** + **H**) and ^18^FDG-PET (not shown) acquired 2 months after treatment showed a near complete regression of the segment 8 lesion and partial response of the segment 4 lesion. Recent follow-up treatment (not shown) consisted of additional ^166^Ho radioembolization of segment 4 and superselective in segment 3. Segment 8 lesion is still in (near) complete remission

Two exemplary clinical cases, illustrating the clinical workflow, are summarized in Figs. 2 and 3 showing an hepatocellular carcinoma and a metastatic intrahepatic cholangiocarcinoma, respectively.

## Radiation Safety

As for any procedure that involves the use of radioactive material, the radiation exposure for personnel should be reduced as much as possible based on the ALARA (as-low-as-reasonably-achievable) principal. During treatment, measurements indicated that the additional radiation exposure to staff caused by the ^166^Ho microspheres procedure is negligible compared to the scattered X-rays from the X-ray tube prior and throughout the procedure [19]. Similar to ^90^Y procedures, precautionary measures, such as the use of a new microcatheter for each injection position and a fluid-absorbing drape should be considered in order to prevent radioactive contamination. Regulation concerning treatment administration and the release of the angiography suite after a ^166^Ho treatment vary between centers and countries. Unforeseen ^166^Ho radioactive contaminations may be more easily detected than ^90^Y microspheres, because of the primary gamma photon emitted by ^166^Ho. Depending on the amount of administered therapeutic activity, patients can be released after treatment with minimal contact restrictions (2 days), based on reduction of radiation by distance and time and in consensus with the instructions by the Nuclear Regulatory Commission for patients with permanent implants. 48 h after infusion, exposure rate and activity excretion have been assessed. Exposure rate at discharge, assessed in 15 patients, was 26 μSv/h, which, extrapolated to a whole liver dose of 60 Gy, would lead to a total effective dose equivalent < 5 mSv [20]. Renal and intestinal ^166^Ho activity excretion was found in all four cases under investigation, independent of the activity of the injected microspheres. The highest total excretion fraction was 0.005% of the injected activity with intestinal excretion being lower than renal excretion [21]. Bakker et al. [22]*,* assessing 1-h blood plasma and 24-h urine, found the median percentage of ^166^Ho compared to the total amount injected to be 0.19% and 0.32%, respectively.

## Clinical Studies on ^166^Ho Radioembolization

**Fig. 4:**
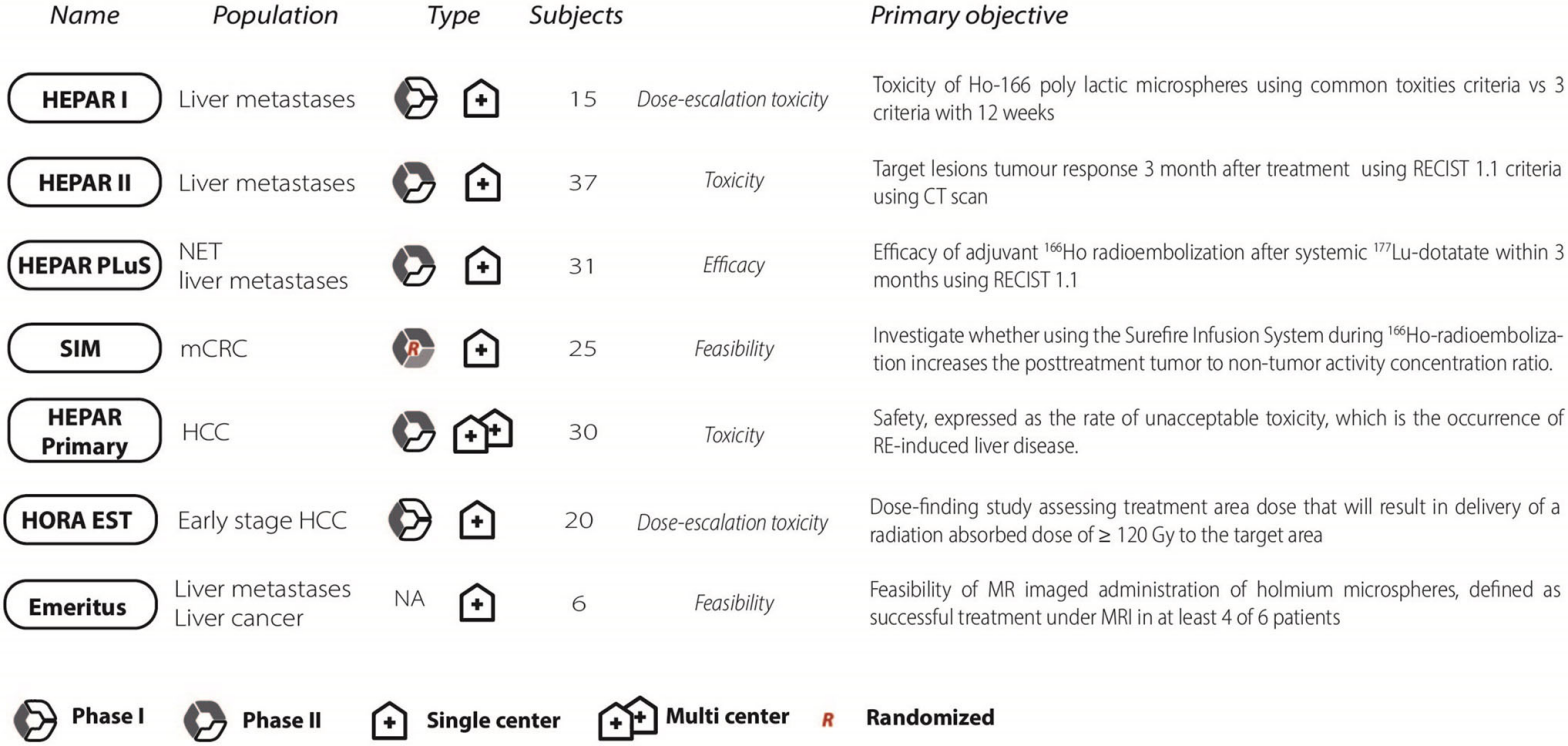
Summary of the clinical studies on ^166^Ho radioembolization completed between 2009 and 2021

From 2009, eight clinical studies using ^166^Ho microspheres for radioembolization have been carried out. Type of study, patients’ population, study phase and design, and primary objective are summarized in Fig. 4. Six other studies, mainly exploring the additional value of individualized treatment are currently in preparation. The findings regarding the primary end-point of the prospective studies completed within 2021 are summarized in Table 4. The first study in humans, a dose escalation study, identified the maximum tolerated dose for ^166^Ho radioembolization at 60 Gy, using the current MIRD method [23]. In addition, it was demonstrated that in vivo dosimetry was feasible by both SPECT and MRI imaging [7]. Subsequently, a phase II study investigated ^166^Ho radioembolization efficacy [24]. A total of 73% of the study population showed complete response, partial response or stable disease at three-month follow-up, with a median overall survival of 14.5 months, confirming safety and showing efficacy. Another phase II study showed that additional ^166^Ho radioembolization after peptide receptor radionuclide therapy in patients with metastatic liver neuroendocrine neoplasms is safe and efficacious [25]. Specifically, 43% of patient population achieved an objective response in the treated volume, according to the per-protocol analysis. In nine patients suffering from hepatocellular carcinomas, Radosa et al. [26] showed that ^166^Ho radioembolization is a feasible and safe treatment option with no significant hepatotoxicity. At six-month follow-up, 89% of patients showed either a complete response, partial response or stable disease. A within-patient randomized study aiming at assessing whether the use of an anti-reflux catheter improves tumor targeting for colorectal cancer patients treated with ^166^Ho radioembolization confirmed efficacy and toxicity findings of previous studies. Laboratory toxicity was reported for 14% of the patients, while clinical toxicity was found in 19%. One patient (5%) died due to radioembolization-induced liver disease. Median overall survival was 7.8 months. At a tumor-level, a significant dose–response relationship was established with mean tumor-absorbed dose in tumors with complete metabolic response 138% higher, on average, than in progressive tumors (222 Gy vs. 103 Gy, respectively). [27]. To conclude, a phase II study assessing toxicity profile of ^166^Ho in patients with hepatocellular carcinoma reported unacceptable toxicity in 10% of the treated patients, but no cases of radioembolization-induced liver disease [28]. Additionally, target liver lesions with complete or partial response were found to be 54% and 84% at three- and six-month follow-up, respectively. Median overall survival was 14.9 months. An observational retrospective study recently started (RECORD), aims at further describe the general safety and clinical performance of ^166^Ho microspheres, with specific attention to outcomes per tumor origin.

**Table 4:** Summary of the published clinical findings deriving from the prospective studies completed so far

References	Study	*N*^a^	Lesion type	Main finding
Reinders-Hut et al. [28]	HEPAR primary	31	HCC (87% multifocal disease)	^166^Ho-radioembolization is a safe treatment option for HCC patients with
				Unacceptable toxicity related to study treatment occurred in 10% of patients
				Complete or partial response for:
				54% of the target liver lesions at 3-month follow-up
				84% of the target liver lesions at 6-month follow-up
				Median overall survival was 14.9 months
Van Roekel et al*.* [27]	SIM	21	Liver metastases (mCRC)	Between anti-reflux and standard microcatheter
				No difference in tumor targeting
				No difference in infusion efficiency
				No influence on the dose–response rate
				Confirmed safety and efficacy in mCRC
Braat et al. [25]	HEPAR PLuS	30	Liver metastases (NET)	^166^Ho radioembolization, as an adjunct to peptide receptor radionuclide therapy is safe and efficacious, with
				Response (complete or partial) in the liver, according to RECIST 1.1
				43% at 3 months
				47% at 6 months
				Acceptable toxicity
				No loss in quality of life
Prince et al. [24]	HEPAR II	37	Liver metastases (different origins)	^166^Ho radioembolization-induced a tumor response and acceptable toxicity profile in salvage patients with
				Complete response, partial response or stable disease of the target lesion obtained in 73% of population at 3-month follow-up
				Median overall survival of 14.5 months
Smits et al. [23]	HEPAR I	15	Liver metastases (different origins)	The maximum tolerated radiation dose was identified as 60 Gy (averaged over the perfused volume)
				Stable disease or partial response regarding target lesions achieved:
				In 93% population at 6-week follow-up
				In 64% population at 12-week follow-up

*T/N* tumor to non-tumor ratio, *RECIST* response evaluation criteria in solid tumors, *HCC* hepatocellular carcinoma, *MCRC* metastatic colorectal cancer, *NET* neuroendocrine tumor

^a^Number of subjects included in the referred article analyzing the mentioned study

## Future Prospective

Many possibilities offered by ^166^Ho liver radioembolization are still to be exploited, especially in clinical practice. Here, the three main directions following from current research are summarized.

### Individualized Treatment

Personalized medicine is the Holy Grail that health care providers would like to reach in the near future to optimize patients’ treatment. In the frame of ^166^Ho radioembolization, this means establishing dose thresholds for patient selection and treatment planning. The definition of robust dose–response values, combined with the use of partition modeling, makes ^166^Ho the desired isotope when it is preferred to perform scout and treatment procedures using the same particle and for quantitative imaging by SPECT or MRI. While retrospective analysis on a dose–response relationship have been recently published [18, 29], prospective studies are currently in preparation. In particular, the recently registered iHEPAR study focuses on assessing the safety of dosimetry-based individualized treatment planning, which has the potential of improved treatment outcomes. However, individualized treatment planning inherently leads to treatment doses that deviate from the currently approved “one-size-fits-all” approach (i.e., 60 Gy average absorbed dose for all patients). Therefore, safety of individualized ^166^Ho radioembolization will be evaluated first to validate safety and confirm safety thresholds.

### Dual Isotope

**Fig. 5:**
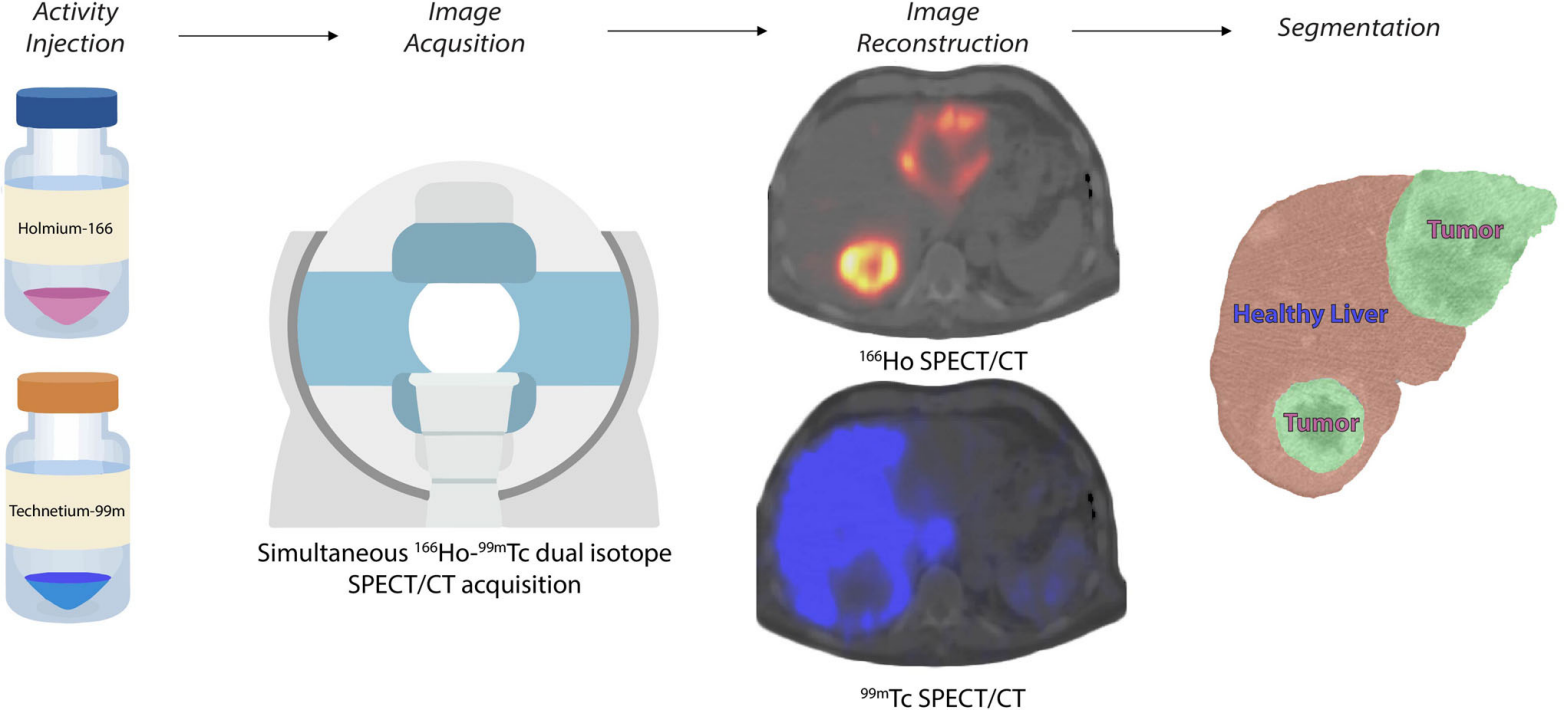
Dual isotope workflow. Firstly, ^166^Ho microspheres are injected (during either the scout or the treatment procedure), lodging primarily in the tumorous tissues. Additionally, ^99m^Tc-stannous phytate is injected on the SPECT table, accumulating in the Kupffer cells representing the healthy liver tissue. Then a conventional SPECT/CT is acquired that simultaneously acquires two isotopes (^166^Ho and ^99m^Tc), after which the images are reconstructed correcting the reciprocal scatter caused by the concomitant presence of the two isotopes. These reconstructions are intrinsically registered and thus can be used to automatically define treated tumors and healthy liver avoiding segmentation and registration of a separately acquired CT, which is time-consuming and prone to error

The possibility to simultaneously use two isotopes to identify healthy liver and tumorous tissue was firstly suggested by Lam et al*.* [30]. A protocol including ^166^Ho scout for treatment simulation and technetium-99m (^99m^Tc) stannous phytate (accumulating in the healthy liver) for healthy liver delineation was proposed to allow for automatic healthy liver segmentation (see Fig. 5). This would avoid the definition of tumor and non-tumorous liver segmentation and registration of a separately acquired contrast enhanced CT or MRI, a time-consuming and prone-to-error task, which is currently necessary to apply the partition modeling enabling personalized activity calculation. The feasibility of this protocol was proved by van Rooij et al*.* [31] using a phantom study and a proof-of-concept clinical case. For a high accuracy in both ^166^Ho and ^99m^Tc reconstruction, they suggested a ^166^Ho:^99m^Tc activity ratio of 5:1. In a phantom experiment, this yielded to a reduction of quantitative ^166^Ho activity recovery by 10% due to the presence of ^99m^Tc. The possibility to use the dual isotope protocol in a clinical setting without hampering the ^166^Ho dosimetry has been demonstrated on 65 clinical procedures [32]. The impact of different ^99m^Tc activity on ^166^Ho quantitative reconstructions and the best method to automatically segment the healthy liver are currently under investigation.

### ^166^Ho Radioembolization Under MRI Guidance

The advantages of ^166^Ho being paramagnetic are not limited to the possibilities to perform quantitative analysis regarding ^166^Ho dosimetry after the treatment. It also enables an MR guided intratumoral ^166^Ho microspheres injection. With the possibility to perform three-dimensional visualization of the tumor, a controlled intratumoral needle placement and visual monitoring of the resulting distribution, it offers for a promising improvement of intratumoral holmium treatment [33]. However, further investigation and fine-tuning of the technique is required to make this method suitable for clinical use.

## Conclusion

Since their introduction as an alternative to ^90^Y microspheres, ^166^Ho microspheres showed unique imaging properties. Additionally, using the ^166^Ho microspheres for both pretreatment and treatment has the benefit of improving the intrahepatic distribution prediction in comparison with current clinical standard. The combination of these features would enable a better patient selection and individualized treatment planning, paving the way to personalized medicine. To this purpose, safety and efficacy dose thresholds should be further investigated, together with the possibility to fully automatize the segmentation and registration processes necessary for adoption of partition modeling for activity calculation.

## Abbreviations

^166^Ho: Holmium-166
^90^Y: Yttrium-90
^99m^Tc: Technetium-99m
CE: Conformitè Europëenne
CT: Computed tomography
PLLA: Poly-l-lactic acid
MAA: Macro aggregated albumin
MIRD: Medical internal radiation dose
MRI: Magnetic resonance imaging
SPECT: Single-photon emission computed tomography
